# Detecting prokaryote-specific gene and other bacterial signatures in thrombi from patients with acute ischemic stroke

**DOI:** 10.1186/s12959-024-00583-x

**Published:** 2024-01-23

**Authors:** Xiaoke Wang, Jie Gao, Yantong Chen, Xiaohao Zhang, Zhengze Dai, Qiliang Dai, Mengna Peng, Lulu Xiao, Xuerong Jia, Haodi Cai, Tao Mou, Xiang Li, Gelin Xu

**Affiliations:** 1 Department of Neurology, Affiliated Jinling Hospital, Medical School of Nanjing University, 305# East Zhongshan Road, 210002 Nanjing, Jiangsu China; 2 Department of Neurology, Shenzhen Second People’s Hospital, Shenzhen, Guangdong China; 3 Department of Neurology, First Affiliated Hospital of Shenzhen University, Shenzhen, Guangdong China; 4 Department of Neurology, Fourth Affiliated Hospital, Nanjing Medical University, Nanjing, Jiangsu China; 5 Department of Neurology, University Medical Center Hamburg-Eppendorf, Martinistraße 52, Hamburg, Germany

**Keywords:** Bacteria, Thrombus, Ischemic stroke, DNA, Endovascular treatment

## Abstract

**Background and purpose:**

Microbial infection has been associated with thrombogenesis. This study aimed to detect bacterium-specific genes and other signatures in thrombi from patients with acute ischemic stroke and to relate these signatures to clinical characteristics.

**Methods:**

Blood samples were collected before thrombectomy procedures, and thrombus samples were obtained during the procedure. Identification and classification of bacteria in the samples were accomplished using 16 S rRNA gene sequencing. Bacterium-specific structures were observed with transmission electron microscopy. Bacterium-specific biomarkers were detected through immunohistochemical staining.

**Results:**

16 S rRNA gene was detected in 32.1% of the thrombus samples from 81 patients. *Bacillus* (0.04% vs. 0.00046%, *p* = 0.003), *Parabacteroides* (0.20% vs. 0.09%, *p* = 0.029), *Prevotella* (1.57% vs. 0.38%, *p* = 0.010), *Streptococcus* (1.53% vs. 0.29%, *p* = 0.001), *Romboutsia* (0.18% vs. 0.0070%, *p* = 0.029), *Corynebacterium* (1.61% vs. 1.26%, *p* = 0.026) and *Roseburia* (0.53% vs. 0.05%, *p* = 0.005) exhibited significantly higher abundance in thrombi compared to arterial blood. Bacteria-like structures were observed in 22 (27.1%), while whole bacteria-like structures were observed in 7 (8.6%) thrombi under transmission electron microscopy. Immunohistochemical staining detected bacterium-specific monocyte/macrophage markers in 51 (63.0%) out of 81 thrombi. Logistic regression analysis indicated that alcohol consumption was associated with a higher bacteria burden in thrombi (odds ratio = 3.19; 95% CI, 1.10–9.27; *p* = 0.033).

**Conclusion:**

Bacterial signatures usually found in the oral cavity and digestive tract were detected in thrombi from patients with ischemic stroke. This suggests a potential involvement of bacterial infection in the development of thrombosis. Long-term alcohol consumption may potentially enhance this possibility.

**Supplementary Information:**

The online version contains supplementary material available at 10.1186/s12959-024-00583-x.

## Introduction

Bacterial infections have been associated with thrombotic events directly or indirectly [1]. Elevated levels of serum antibodies against *Aggregatibacter actinomycetemcomitans*, *Porphyromonas gingivalis*, and *Helicobacter pylori* have been linked to an increased risk of stroke [2]. Gut microbial ligands could potentially promote atherogenesis and thrombogenesis by activating Toll-like receptors [3, 4]. Periodontitis and tooth loss, which are usually caused by oral microbiota dysbiosis [5], have been associated with a higher risk of stroke [6]. Exposure to multiple microorganisms, known as infectious burden, may result in greater cardiovascular risks compared to exposure to a single microorganism [7].

Recent studies have shown various bacteria within atherosclerotic plaques and thrombi, indicating their potential involvement in related conditions. The use of 16 S ribosomal DNA (rDNA) analysis has allowed for the detection of microbial signatures from both oral and gut microbiota within atherosclerotic plaques [8, 9]. Oral bacteria have also been detected within thrombi obtained from patients with acute myocardial infarction and peripheral vascular disease [10–12]. Periodontal bacteria, including *Streptococcus mutans*, *Prevotella intermedia*, and *Porphyromonas gingivalis*, have been found in the valvular tissue of patients with cardiovascular diseases [13].

The scarcity of available biological samples has limited the investigation of bacterial signatures in cerebral artery thrombus. Recent technical progress and accumulating evidence have facilitated the clinical application of endovascular thrombectomy for the treatment of acute ischemic stroke. This procedure has allowed for the retrieval of a significant number of cerebral artery thrombi, making it technically feasible to conduct component analysis on these thrombi. In light of these developments, this study aimed to investigate the microbial community signatures and assess the diversity of bacteria within thrombi obtained from patients with acute ischemic stroke.

## Materials and methods

### Patients

During the period from January 1, 2018, and October 31, stroke patients who underwent endovascular recanalization treatment were recruited from Jinling Hospital for this study. The Ethic Review Board of Jinling Hospital approved the study protocol, and all participants provided informed consent before enrollment.

The inclusion criteria for the study involved patients who were diagnosed with acute ischemic stroke and underwent mechanical endovascular thrombectomy. Patients were excluded if they had a known diagnosis of bacteremia or septicemia at the time of enrollment or if no thrombus was successfully retrieved during the endovascular procedure.

### Stroke evaluation and endovascular treatment

The severity of stroke in the enrolled patients was assessed using the National Institutes of Health Stroke Scale (NIHSS). Subtypes of ischemic stroke were determined according to the Trial of Org 10,172 in Acute Stroke Treatment (TOAST) criteria [14]. Successful reperfusion following the endovascular thrombectomy procedure was defined as a modified Thrombolysis in Cerebral Infarction Score (mTICI) of 2b/3 [15].

### Detection of bacterial signatures

Each thrombus was promptly divided into 3 segments after being retrieved. One segment was placed in a pyrogen-free Eppendorf tube for nucleic acid sequencing. Another segment was fixed in 4% paraformaldehyde for immunohistochemical examination, and the third one was fixed in 2.5% glutaraldehyde for electron microscopic scanning. Arterial blood and venous blood samples were obtained from the patients before the endovascular procedure, and stored in pyrogen-free Eppendorf tubes. These blood samples served as control samples for comparative analysis. All samples were stored at -80 °C before tests.

DNA extraction from the collected samples was performed using the QIAamp DNA Blood Mini Kit (Qiagen, Germany) following the manufacturer’s manual. After ethanol precipitation, DNA samples were resuspended in double-distilled water and stored at -80 °C until further use. All DNA extraction procedures were conducted under a class II biologic safety cabinet.

Universe primers (341 F and 806R) were used for PCR amplification of the V3-V4 region of the bacterial 16 S rRNA gene. PCR reactions were performed in a 20 µL mixture, which included a 10$\times$polymerase mix (Life Technologies, Carlsbad, CA), 10 µM concentrations of the forward and reverse primers, and 25 ng of template DNA. The amplicon sequencing libraries were prepared from the PCR products and sequenced on an Illumina HiSeq platform, generating 250-bp paired-end reads. Negative controls were included to account for potential contamination during the DNA extraction and amplification process. These controls consisted of empty sterile storage tubes that underwent the same procedures and used the same reagents as the blood samples. This helped identify and exclude any potential contaminants. Thrombi that were successfully sequenced using high-throughput sequencing methods and showed the presence of prokaryote-specific DNA was defined as Prokaryote-Specific DNA (+).

For transmission electron microscopic observation, the thrombus segments were prepared into thin sections using an ultra-microtome (UC7, Leica, Germany). These sections were then post-stained with uranyl acetate and lead citrate (Servicebio, China). The electron micrographs were taken with a Hitachi HT7800/7900 transmission electron microscope operating at 80 kV.

For immunohistochemistry, the thrombus segments were cut into 5${\upmu }\text{m}$thick microns sections using a manual microtome (RM2016, Leica, Germany). The activity of the bacterium-specific CD14 marker was assessed using a 1:100 dilution of the SP192 clone antibody (ab183322, Abcam, Britain) applied to the thrombus sections. Following antibody incubation, diaminobenzidine (G1211, Servicebio, China) was used as a chromogen to visualize the presence of CD14.

### Statistical analysis

The raw data obtained from 16 S rDNA sequencing were processed to generate operational taxonomic units (OTUs) at a 97% identity threshold using UPARSE. Taxonomy was assigned using the Ribosomal Database Project (RDP) as the reference database. The α- and β-diversity indices were calculated based on the rarefied OTU counts using the Qiime program. Statistical comparisons of α-diversity indices, including the Chao1 index, observed_species index, Shannon index, and Simpson index, were conducted using the Wilcoxon rank sum test and Kruskal-Wallis test.

Quantitative variables were reported as mean ± SD or median (interquartile range) and compared with Student’s t-test or Mann–Whitney *U* as appropriate. Categorical variables were reported as frequency (proportion), and compared with the χ^2^ test or Fisher exact test as appropriate. Similarities of bacterial species between thrombus and blood samples were analyzed using the R package “ade4”. Distinguishing microbial features between groups were identified using the linear discriminant analysis (LDA) effect size (LEfSe) method with an alpha cutoff of 0.05 and an effect size cutoff of 2.0. Differential abundance analysis was performed using the Kruskal-Wallis and Wilcoxon rank sum test. The association between bacteria burden and characteristics was evaluated using multivariable logistic regression. Statistical significance was defined as a *P*-value less than 0.05 for two-tailed tests. All statistical analyses were performed using SPSS version 22.0 (IBM, Armonk, NY).

## Results

**Fig. 1:**
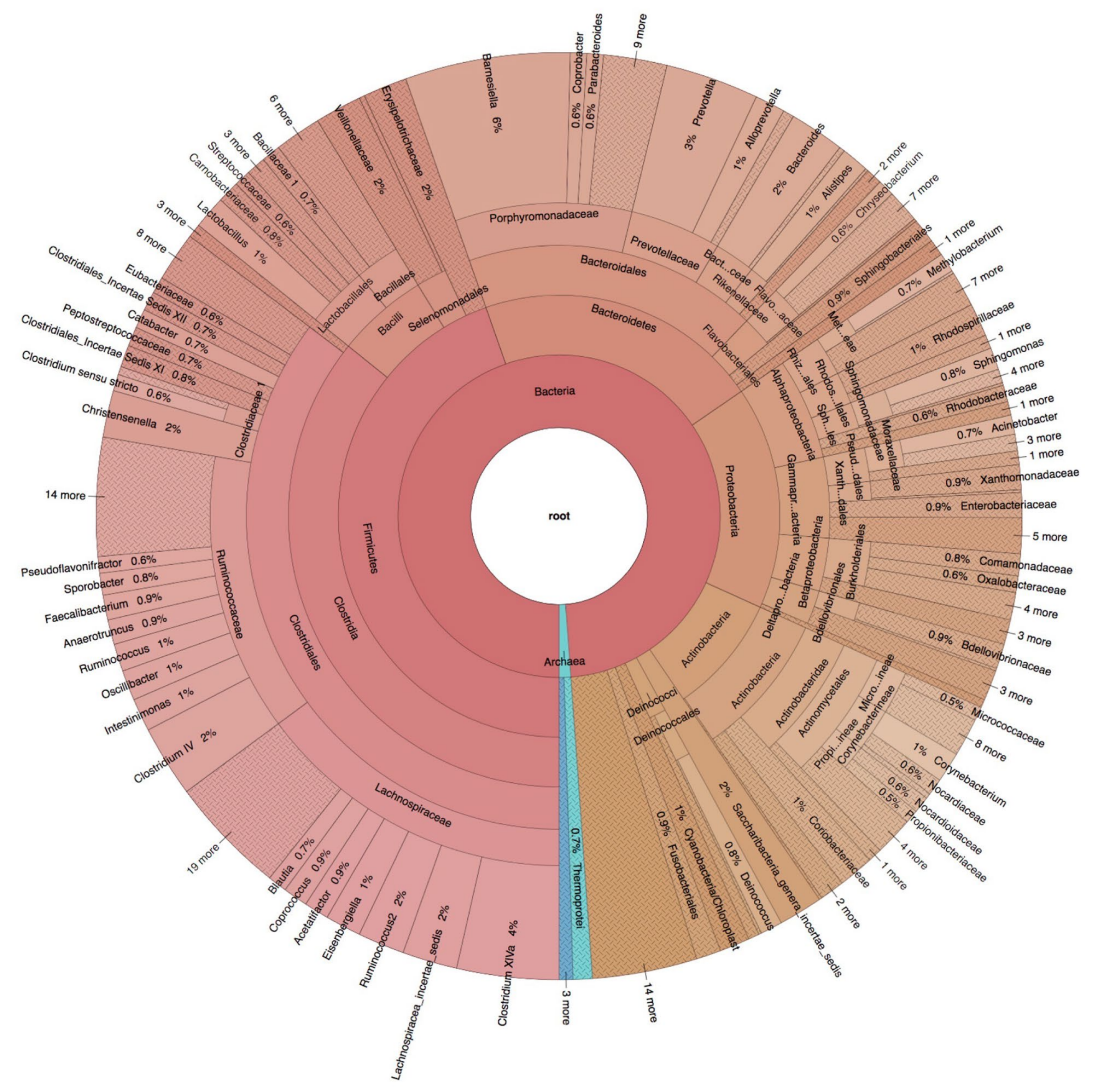
The Krona chart of the bacteria in thrombus by 16 S rRNA gene sequences

A total of 81 patients with acute ischemic stroke were recruited, with a median age of 70 years and 52 (64.2%) of them being male. The median procedure time was 80 min, with an interquartile range (IQR) of 57 to 113. The median preoperative National Institute of Health Stroke Scale (NIHSS) score was 17 (IQR, 13 to 24). Among the 81 thrombus samples collected from the patients, the prokaryote-specific 16 S rRNA gene was detected in 26 (32.1%) samples. More detailed patient characteristics are shown in Table 1. Further analysis revealed a diverse microbial composition, with a total of 1042 operational taxonomic units (OTUs) belonging to 296 genera, 141 families, 72 orders, 40 classes, and 21 phyla detected in these 26 thrombi. Among the identified microbes, the two most abundant bacterial phyla observed in the thrombi were *Firmicutes* and *Bacteroidetes* (Fig. 1).

**Table 1:** Patient Characteristics According to Prokaryote-Specific DNA in Thrombi

	Prokaryote-Specific DNA		*p* value
	+ *n* = 26	− *n* = 55	
Age, media (IQR)	72 (65–75)	69 (57–78)	0.46
Male gender, n (%)	20 (76.9)	32 (58.2)	0.10
BMI, media (IQR)	23.9 (21.5–25.4)	24.1 (23.2–26.1)	0.55
Hypertension	16 (61.5)	37 (67.3)	0.61
Diabetes	8 (30.8)	11 (20.0)	0.29
AF	16 (61.5)	33 (60.0)	0.62
CAD	9 (34.6)	12 (21.8)	0.22
Prior stroke	7 (26.9)	11 (20.0)	0.48
Smoking	11 (42.3)	13 (23.6)	0.09
Alcohol drinking	10 (38.5)	9 (16.4)	0.03*
Intravenous alteplase use	3 (11.5)	9 (16.4)	0.74
NIHSS score, media (IQR)	16 (11–22)	18 (14–25)	0.08
**Stroke sub-type, n (%)**			0.19
LAA	7 (26.9)	13 (23.6)	
Cardioembolic	16 (61.5)	29 (52.8)	
Others or uncertain	3 (11.6)	13 (23.6)	
HDL, mmol/L, media (IQR)	1.18 (0.84–1.40)	1.11 (0.87–1.38)	0.79
LDL, mmol/L, media (IQR)	2.68 (1.44–3.06)	2.49 (2.01–2.98)	0.98
TG, mmol/L, media (IQR)	0.68 (0.53–1.06)	0.96 (0.67–1.26)	0.08
TC, mmol/L, media (IQR)	3.89 (3.21–4.87)	4.26 (3.43–4.55)	0.98
Leukocyte, ×10^9^/L, media (IQR)	10.01 (8.06–13.06)	9.39 (7.88–12.60)	0.27
CRP, mg/L, media (IQR)	3.55 (0.50-13.09)	9.9 (3.60–23.50)	0.17
**Occlusion site, n (%)**			0.75
ICA	9 (34.6)	19 (34.5)	
MCA	15 (57.7)	28 (50.9)	
Vertebrobasilar	2 (7.7)	8 (14.5)	
Procedure time, media (IQR)	66 (55–95)	85 (58–120)	0.14
Thrombus fragmentation, n (%)	9 (34.6)	30 (54.5)	0.09
Successful reperfusion, n (%)	24 (92.3)	42 (76.4)	0.13

BMI, body mass index; AF, Atrial fibrillation; CAD, coronary atherosclerosis disease; NIHSS, National Institute of health stroke scale; LAA, large-artery atherosclerosis; CRP, C-reactive protein; HDL, high density lipoprotein cholesterol; LDL, low density lipoprotein cholesterol; TG, Triglycerides; TC, Total cholesterol; ICA, internal carotid artery; MCA, middle cerebral artery

**Fig. 2:**
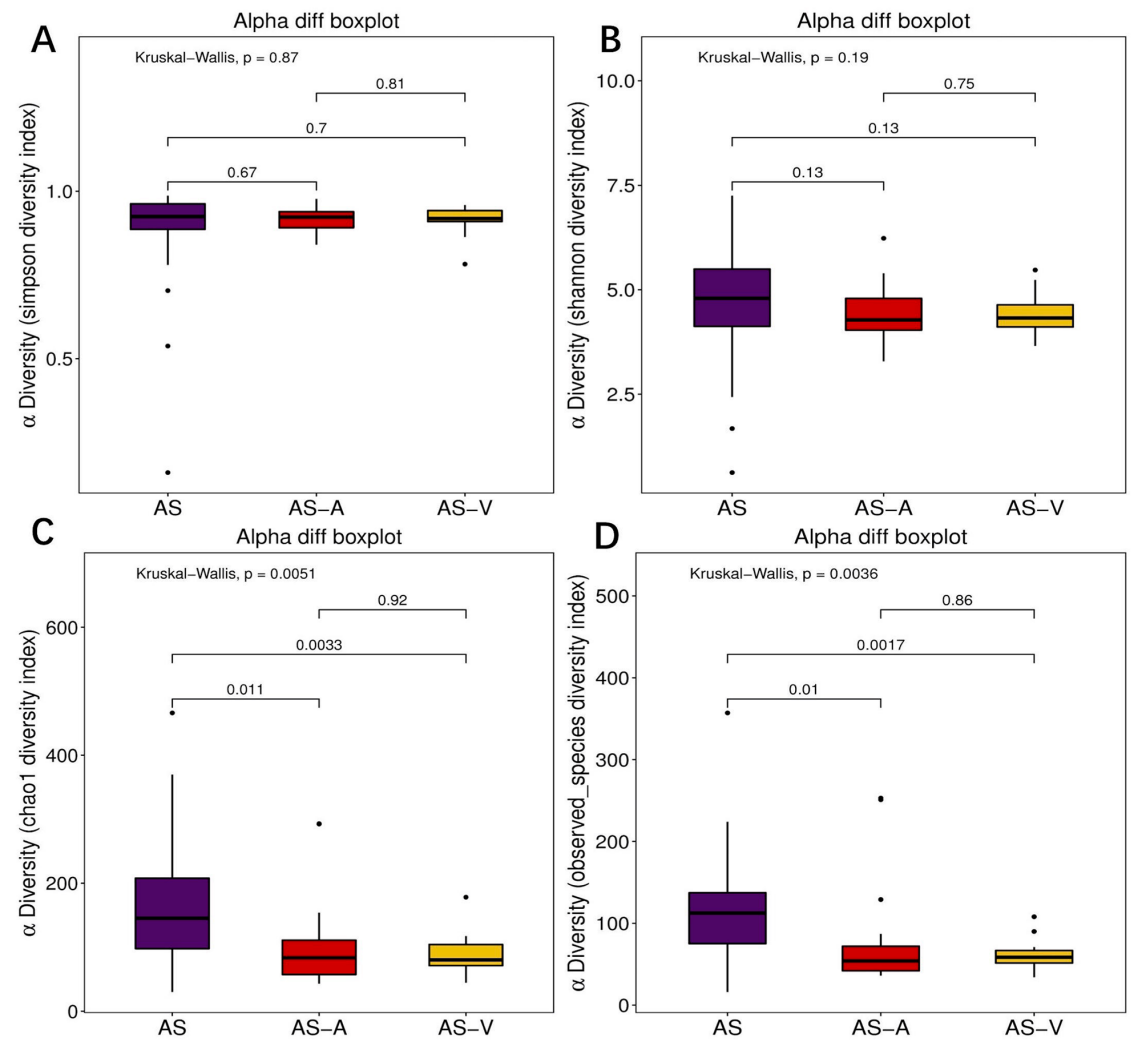
The α -diversity diagram of bacteria in thrombus and blood samples. (**A**) Simpson index; (**B**) Shannon index; (**C**) Chao1 index; (**D**) Observed species index. AS, thrombus; AS-A, arterial blood; AS-V, venous blood; *P < 0.05

Microbial diversity was compared between thrombus and blood samples obtained from the same patients. No significant differences were found when analyzing the Simpson index and Shannon index, indicating similar levels of diversity between thrombus and arterial blood samples. Both the observed species index and Chao1 index were higher in thrombi compared to arterial blood. Furthermore, the observed species index and Chao1 index in thrombi were significantly higher than those observed in venous blood (Fig. 2). The higher number of OTUs in the thrombi suggested a greater relative abundance of bacteria within the thrombi compared to the blood samples. Additional analyses, such as ANOSIM (*p* = 0.002, Supplementary Fig. 1A) and PCoA (*p* = 0.001, Supplementary Fig. 1B) demonstrated significant differences in bacterial characteristics between thrombi and both arterial and venous blood samples.

**Fig. 3:**
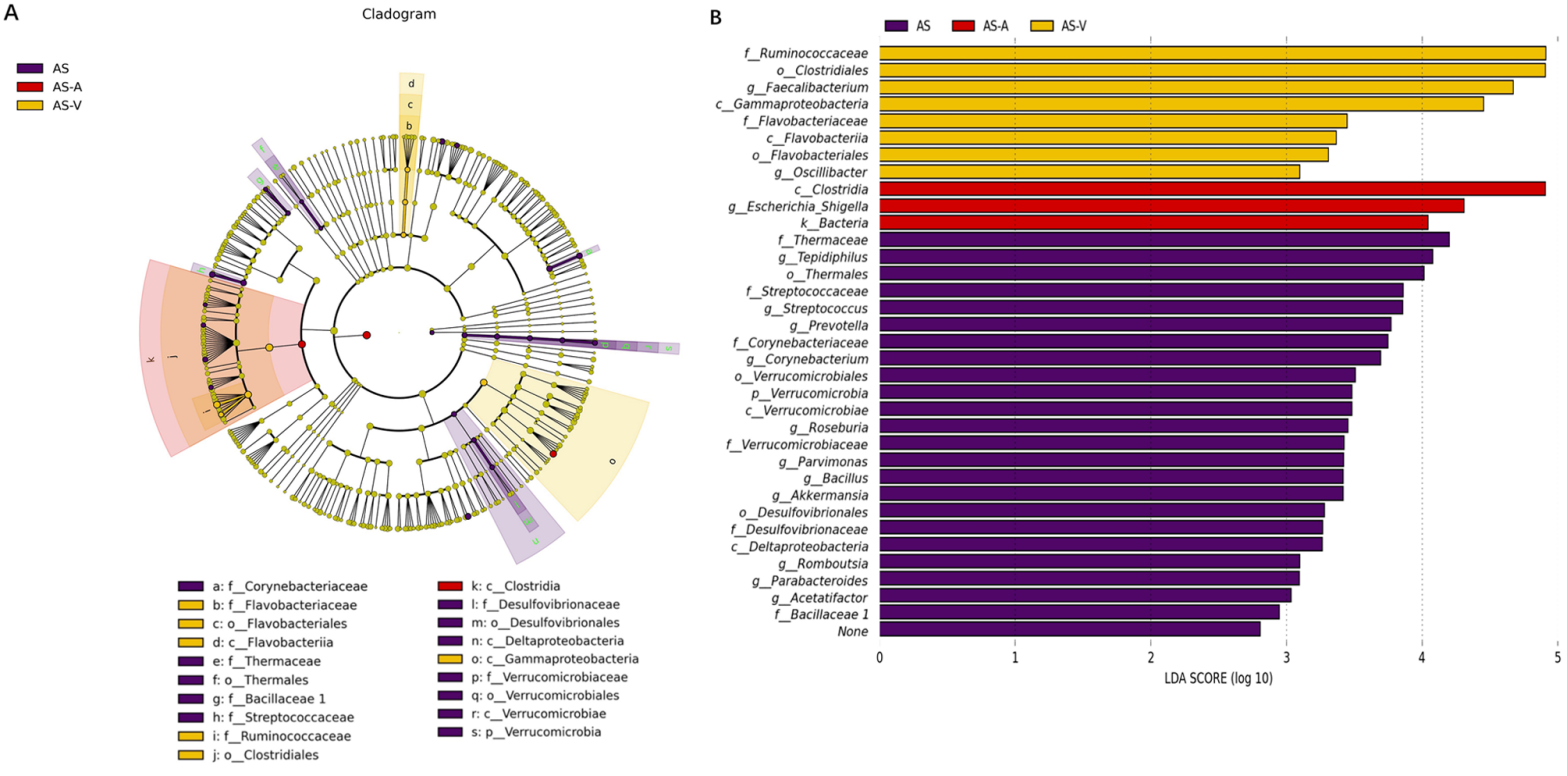
Differentially abundant taxa. Linear discriminant analysis (LDA) effect size was performed on the microbial communities of the thrombus and blood samples. (**A**) Taxonomic cladogram of the microbiota. (**B**) Histogram of the LDA scores for significantly discriminant taxa between thrombus and blood samples

LEfSe analysis revealed significant taxonomic differences in thrombus samples, highlighting specific bacterial species that were enriched in the thrombus environment. Notably, *Thermaceae* (LDA = 4.2, *p* = 0.037), *Streptococcacea* (LDA = 4.2, *p* = 0.037), *Corynebacteriaceae* (LDA = 3.8, *p* = 0.025), and *Verrucomicrobiaceae* (LDA = 3.4, *p* = 0.036) were identified as significantly bacterial taxa in thrombi (Fig. 3). In addition to taxonomic differences, the relative abundance of specific bacterial species exhibited significant variations between thrombi and arterial blood samples. *Bacillus* (0.04% vs. 0.00046%, *p* = 0.003), *Parabacteroides* (0.20% vs. 0.09%, *p* = 0.029), *Prevotella* (1.57% vs. 0.38%, *p* = 0.010), *Streptococcus* (1.53% vs. 0.29%, *p* = 0.001), *Romboutsia* (0.18% vs. 0.0070%, *p* = 0.029), *Corynebacterium* (1.61% vs. 1.26%, *p* = 0.026) and *Roseburia* (0.53% vs. 0.05%, *p* = 0.005) exhibited higher relative abundances in thrombi compared to arterial blood (Table 2). Conversely, the relative abundance of *Faecalibacterium* (9.15% vs. 17.07%, *p* = 0.008) and *Escherichia/Shigella* (4.09% vs. 8.26%, *p* = 0.003) in thrombi were lower than that in arterial blood. Supplementary Table 1 provides additional information on relative abundance differences between thrombi and venous blood samples.

**Table 2:** Prevalence and Mean Relative Abundance of Significant Genus

Genus	Prevalence, n (%)	Mean relative abundance		Fold change	*p* value
		Thrombus	Arterial blood	Thrombus vs. Artery blood	
*Streptococcus*	21 (80.8)	1.53%	0.29%	5.2	0.001
*Prevotella*	19 (73.1)	1.57%	0.38%	4.1	0.010
*Parabacteroides*	13 (50.0)	0.20%	0.09%	2.4	0.029
*Romboutsia*	12 (46.2)	0.18%	0.0070%	26.2	0.029
*Roseburia*	16 (61.5)	0.53%	0.05%	9.7	0.005
*Bacillus*	8 (30.8)	0.04%	0.00046%	86.7	0.003
*Corynebacterium*	22 (84.6)	1.60%	1.26%	1.3	0.026
*Escherichia/Shigella*	25 (96.2)	4.09%	8.26%	0.5	0.003
*Faecalibacterium*	24 (92.3)	9.14%	17.07%	0.5	0.008

**Fig. 4:**
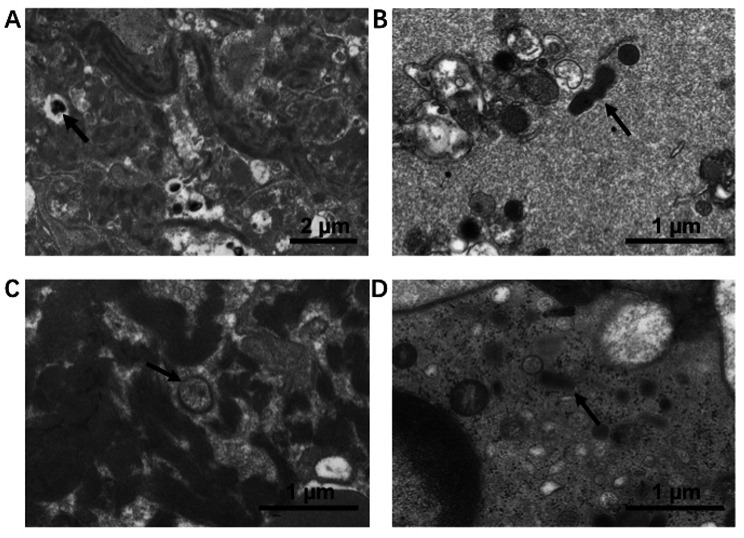
Electron microscopy observation of bacteria in thrombus. (**A**) and (**B**) Coccus were distributed in clusters. (**C**) and (**D**) Bacteria with complete cell wall structure

**Fig. 5:**
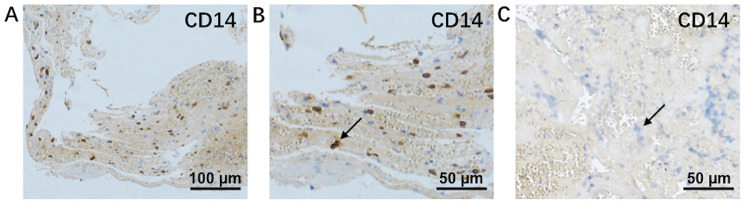
Immunohistochemical staining of CD14. (**A**) and (**B**) originated from the same thrombus. In image (B), the black arrow indicated a positively stained cell. (C) served as the negative control. In image (**C**), the black arrow signified a negatively stained cell

Partial bacteria-like structures were observed in 22 (27.1%) thrombi samples and whole bacteria-like structures were observed in 7 (8.6%) thrombi samples. These observations were made using transmission electron microscopy (Fig. 4). Immunohistochemical staining targeting CD14, a bacterium-specific monocyte/macrophage marker, revealed positive staining in 63.0% of the 81 thrombi samples examined. Figure 5 visually represents the immunohistochemical staining results for CD14 in the thrombi.

The logistic regression analysis revealed a significant association between the presence of prokaryote-specific DNA (+) and alcohol consumption among the patients. 38.5% of the prokaryote-specific DNA (+) patients were found to be alcohol drinkers, compared to 16.4% among those without prokaryote-specific DNA. This difference in alcohol consumption between the two groups was statistically significant (*p* = 0.03). After adjusting for age and sex, multivariable logistic regression analysis indicated that current alcohol drinking was significantly associated with a higher bacteria burden in thrombi (odds ratio = 3.19; 95% CI, 1.10–9.27; *p* = 0.033).

## Discussion

This study analyzed the microbiome in thrombi, arterial blood, and venous blood samples obtained from patients with acute ischemic stroke. The results revealed that several bacterial taxa, including *Bacillus*, *Corynebacterium*, *Parabacteroides, Romboutsia*, *Roseburia, Prevotella*, and *Streptococcus*, exhibited a higher relative abundance in thrombi compared to arterial blood samples. Our findings may suggest a distinct microbial profile within the thrombus compared to the circulating arterial blood.

A wide range of infectious agents have been associated with the development of atherosclerosis [16, 17]. *Streptococcus mitis*, commonly found in the oral cavity, can enter blood flow, adhere to vascular endothelium, induce platelet aggregation, and then form a biofilm that may serve as attachment points for other bacteria [18]. Periodontitis, caused by Gram-negative bacteria, can lead to persistent endotoxemia [19, 20]. It is worth noting that endotoxemia is quite common after periodontal examination, tooth extractions, and even tooth brushing [21, 22]. The gut, which has the greatest variety and number of bacteria in the human body [23], is considered a significant source of endotoxins [24]. Bacteria can enter the bloodstream and reach atherosclerotic plaques through compromised intestinal epithelial membranes or macrophage phagocytosis [25]. Studies have demonstrated the presence of oral and gut bacteria in valvular vegetation, endocardium, and atherosclerotic plaques [8, 13, 26]. These findings indicate the potential involvement of both oral and gut bacteria in the pathogenesis of atherosclerosis.

Accumulating evidence suggests that bacterial infection can accelerate the rupture of atherosclerotic plaque [9, 27]. The debris resulting from these plaque ruptures can then obstruct downstream arteries, potentially leading to cerebral infarction. When a plaque ruptures, the microorganisms contained within it can enter the in-situ thrombus [12]. Additionally, specific species such as *Bacillus cereus* and *Bacillus anthracis* have been found to have the ability to directly initiate blood coagulation and enter the thrombus [28]. These mechanisms further emphasize the potential role of bacterial infections in promoting thrombus formation and its subsequent complications.

Left atrial remodeling plays a significant role in the development of atrial fibrillation (AF) and stroke. One of the key features of left atrial remodeling is the dilation and dysfunction of the left atrium, which can result in blood stasis within the atrial chambers [29]. This stagnant blood flow provides an ideal milieu for bacteria to aggregate and potentially initiate infections. Bacterial interactions with platelet receptors are of particular importance in the context of thrombosis. Lipopolysaccharide, a common component in the outer membrane of Gram-negative bacteria, has the capability to bind to Toll-like receptor 4 located on platelets. This binding triggers platelet activation and aggregation though the MyD88-dependent signaling cascade [30]. Protein disulfide isomerase (PDI) plays a crucial role in coagulation factor modulation and platelet integrin-mediated aggregation by influencing thiol-disulfide exchange processes [31]. Bacterial homologs of PDI, known as bacterial disulfide bond (DSB) proteins, may share a similar potential to activate platelets [32]. Platelet activation can further trigger neutrophil engagement and the release of neutrophil extracellular traps, all of which contribute to the promotion of blood coagulation [33]. Moreover, bacterial DNA has been shown to have heparin-like properties. This bacterial DNA can form antigenic complexes similar to those formed with heparin, resulting in comparable levels of platelet aggregation and thrombosis [34]. This highlights the potential role of bacterial DNA in promoting thrombotic events.

This study established an association between alcohol consumption and the bacterial burden observed in cerebral artery thrombi. Previous research has linked alcohol drinking to the advancement of atherosclerosis [35]. Long-term alcohol consumption has been shown to alter the composition of the intestinal microbiota and compromise the integrity of the intestinal mucosal barrier [36]. Consequently, the increased intestinal permeability may facilitate the entry of pathogens into the bloodstream [37, 38]. Heavy alcohol consumption has been specifically associated with an elevated cardiovascular risk within the subsequent day [39]. These findings suggest that the detrimental effects of alcohol consumption on the intestinal microbiota and mucosal barrier may contribute to an increased bacterial burden in cerebral artery thrombi.

Our study acknowledged a major limitation regarding the 16 S rRNA gene sequencing method employed, which only allowed for the detection of bacterial DNA within the samples but not the live microorganisms or their metabolic activity. The small size of the samples hindered the quantification of bacterial DNA concentration within the thrombi. Additionally, there is a possibility that the observed excessive relative abundance of certain genera within the thrombi could be attributed to phagocytized bacterial components derived from biofilms. These limitations underscore the need for future studies to explore the functional aspects of bacterial involvement in thrombogenesis, beyond mere DNA detection, and to investigate the specific roles played by bacterial components in the process.

The detection of bacterial signatures typically found in the oral cavity and digestive tract within thrombi from patients with ischemic stroke suggests a potential role for bacterial infection in thrombogenesis and an increased risk of ischemic stroke. This finding supports the idea that bacteria present in these regions may contribute to the development of thrombi and subsequent stroke. Moreover, long-term alcohol consumption has been associated with an increased risk of ischemic stroke. This suggests that alcohol drinking may further enhance the risk of bacterial infection-associated thrombogenesis and ischemic stroke. These observations highlight the potential interplay between bacterial infection, thrombogenesis, and the increased risk of ischemic stroke. Further research is needed to better understand the underlying mechanisms and develop effective preventive measures and treatments.

### Electronic supplementary material

Below is the link to the electronic supplementary material.

Supplementary Material 1

## Data Availability

The dataset analyzed during our study is available upon reasonable request to the corresponding author, subject to obtaining the appropriate approvals.
