# Supplementary material for: Detecting prokaryote-specific gene and other bacterial signatures in thrombi from patients with acute ischemic stroke
Source: Thromb J. 2024 Jan 23;22:14. doi: 10.1186/s12959-024-00583-x (PMC10807108; doi:10.1186/s12959-024-00583-x)

**Supplementary Figure legends**

**Figure S1** Thrombus and blood $\beta$-diversity diagram

(A) Anosim analysis of unweighted uniFrac distances for thrombus, artery, and venous blood subjects; (B) Adonis analysis, principal coordinates analysis (PCoA) of the unweighted uniFrac distances for thrombus, artery, and venous blood subjects. The two components explain 12.2% and 6.5% of the variance. A significant separation was found between groups (R^2^ =0.057, *p* < 0.002). Purple, thrombus (n = 26); red, artery blood (n =23); yellow, venous blood (n =19).

**Table S1** The Prevalence and Mean Relative Abundance of Significant Genus

| **Genus** | **Prevalence (%)** | | **Mean relative abundance**  **Thrombus Venous blood** | | | | **Fold change**  **Thrombus vs Venous** | | ***p* value** |
| --- | --- | --- | --- | --- | --- | --- | --- | --- | --- |
| *Streptococcus* | | 21 (80.8%) | | 1.53% | 0.77% | 2.0 | | 0.009 | |
| *Acetatifactor* | | 8 (30.8%) | | 0.12% | 0.00% | - | | 0.011 | |
| *Parabacteroides* | | 13 (50.0%) | | 0.20% | 0.0024% | 85.7 | | 0.014 | |
| *Romboutsia* | | 12 (46.2%) | | 0.18% | 0.02% | 9.3 | | 0.033 | |
| *Roseburia* | | 16 (61.5%) | | 0.53% | 0.0050% | 111.5 | | 0.005 | |
| *Bacillus* | | 8 (30.8%) | | 0.04% | 0.00% | - | | 0.014 | |
| *Corynebacterium* | | 22 (84.6%) | | 1.60% | 0.50% | 3.2 | | 0.022 | |
| *Escherichia/Shigella* | | 25 (96.2%) | | 4.09% | 7.55% | 0.5 | | 0.008 | |
| *Faecalibacterium* | | 24 (92.3%) | | 9.14% | 18.70% | 0.5 | | 0.006 | |

**Figure S1**

**
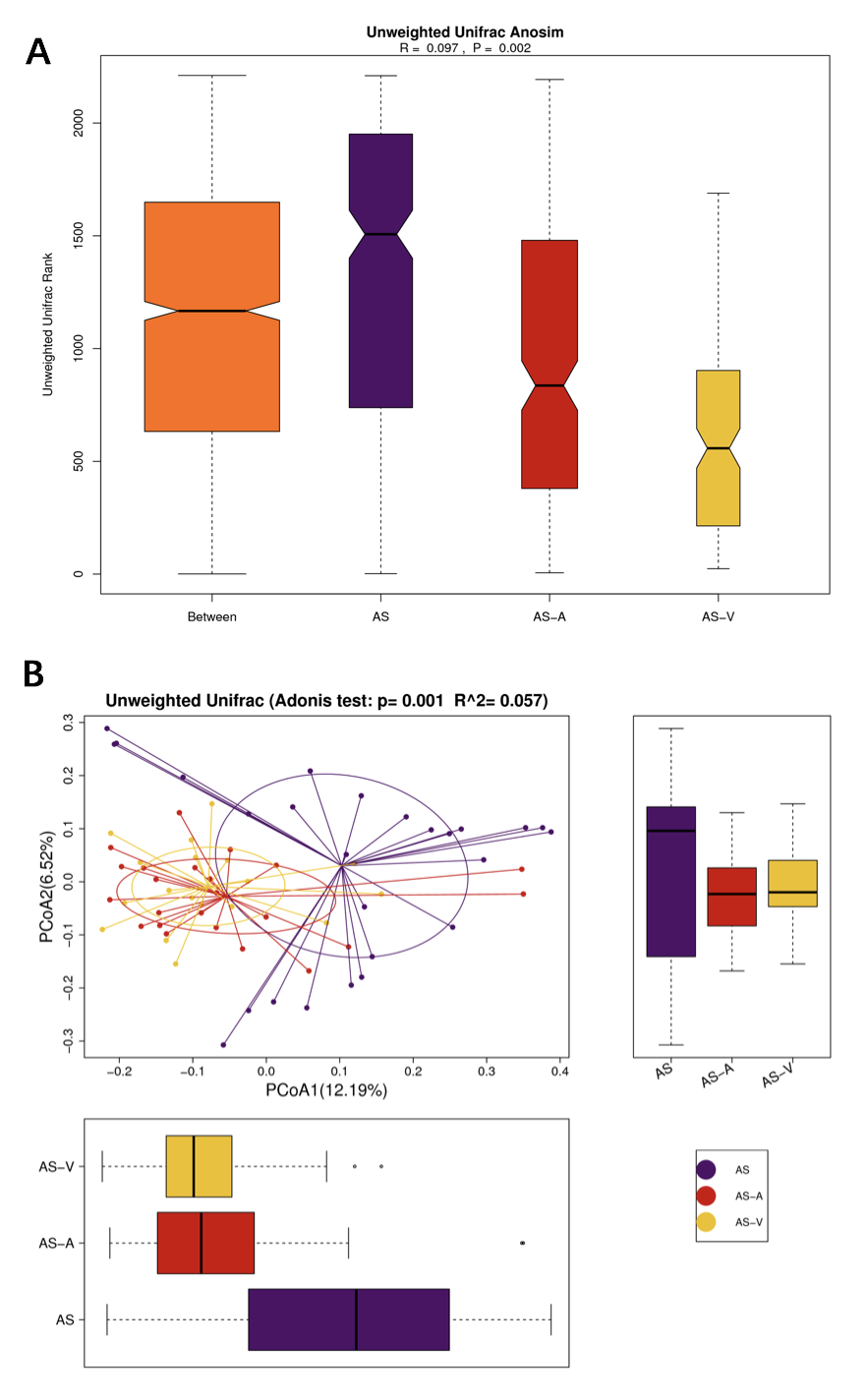
**


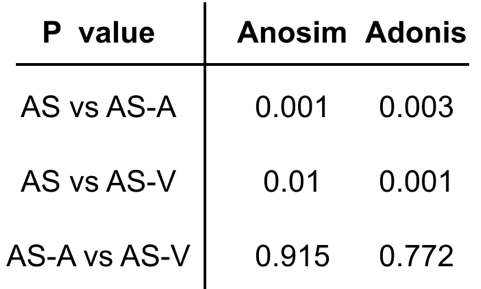

Supplement: Supplementary file 1 — Supplementary Material 1 [file 12959_2024_583_MOESM1_ESM.docx]
